# Real-time CMR combined with cardiopulmonary exercise testing enables integrated assessment of cardiac function during exercise

**DOI:** 10.3389/fcvm.2026.1873004

**Published:** 2026-08-11

**Authors:** Lena Maria Röwer, Duy Tam Vu, Mohamed Ali Goundi, Halima Malik, Pablo Emilio Verde, Dirk Voit, Jens Frahm, Anja Müller-Lutz, Gerald Antoch, Michael Steinmetz, Christian Meierhofer, Dirk Klee, Frank Pillekamp

**Affiliations:** 1 Department of Diagnostic and Interventional Radiology, Medical Faculty and University Hospital Düsseldorf, Heinrich Heine University Düsseldorf, Düsseldorf, Germany; 2 Coordination Centre for Clinical Trials, University Hospital Düsseldorf, Heinrich Heine University Düsseldorf, Düsseldorf, Germany; 3 Max Planck Institute for Multidisciplinary Sciences, Göttingen, Germany; 4 Biomedical Physics, Heinrich Heine University Düsseldorf, Düsseldorf, Germany; 5 CARID, Cardiovascular Research Institute Düsseldorf, University Hospital Düsseldorf, Heinrich Heine University, Düsseldorf, Germany; 6 Department of Pediatric Cardiology and Intensive Care Medicine, University Medical Center, Georg-August-University, Goettingen, Germany; 7 Congenital Heart Disease and Pediatric Cardiology, German Heart Center Munich, Technical University of Munich, Munich, Germany

**Keywords:** cardiac function, cardiac magnetic resonance (CMR) imaging, cardiopulmonary exercise testing (CPET), cardiovascular MRI, Fick principle, image processing computer-assisted, MR-CPET, real-time MRI

## Abstract

**Background:**

Cardiopulmonary exercise testing (CPET) is the gold standard for assessing cardiopulmonary responses to physical stress. Real-time magnetic resonance imaging (RT-MRI) enables continuous cardiovascular imaging during free breathing (FB) and dynamic exercise.

**Objective:**

This study aimed to develop a clinically applicable, easy-to-use MR-CPET protocol using user-friendly postprocessing software and to describe preliminary physiological observations in healthy subjects, forming the basis for future clinical applications.

**Material and Methods:**

Healthy adults (*n* = 12) underwent conventional cine cardiac magnetic resonance imaging (CMR) and cardiac RT-MRI at rest and during submaximal exercise in a 1.5 T MR scanner. The protocol included short-axis cine imaging for volumetry and phase-contrast imaging for aortic flow quantification. CPET was performed using an MR-compatible spirometry and a supine exercise setup enabling continuous measurement of respiratory airflow and gas exchange during image acquisition and simultaneous exercise. Data processing included automated spirometry analysis, temporal synchronization with RT-MRI, and ECG- and respiratory-based binning for volumetric reconstruction. Indexed oxygen consumption (VO₂i) and cardiac index (CI) were used to calculate the arteriovenous oxygen difference (a-vO_2_ diff) according to the Fick equation. Participant comfort was assessed.

**Results:**

The examination was well tolerated, with only minor discomfort related to the spirometry mask. RT-MRI showed strong agreement with conventional cine MRI for left ventricular volumes and ejection fraction at rest. Submaximal exercise significantly increased heart rate and ventilation, with concomitant increases in VO_2_i (201- 309 mL/min/m^2^) and CI (3.38- 4.24 L/min/m^2^), while ventricular volumes slightly decreased and ejection fraction remained unchanged. The calculated a-vO_2_ diff increased from 5.9 to 7.3 mL/dL, indicating enhanced peripheral oxygen extraction.

**Data conclusion:**

Combined CPET and cardiac RT-MRI using the presented user- and participant-friendly setup enables integrated assessment of cardiopulmonary performance and cardiovascular function during submaximal exercise and provides physiologically plausible Fick-derived measures of oxygen transport and utilization.

## Introduction

Cardiopulmonary exercise testing (CPET) is considered the gold standard for assessing the physiological response of the cardiopulmonary system to physical stress and provides comprehensive insight into both normal physiology and exercise tolerance. CPET is widely used as a diagnostic and research tool in adult and pediatric populations as well as in healthy athletes (1–4). Key parameters such as peak oxygen consumption (VO_2_), oxygen pulse, ventilatory efficiency, and anaerobic threshold are commonly used to characterize exercise intolerance and to predict clinical outcomes across a broad spectrum of cardiovascular and pulmonary diseases (1, 4, 5). However, despite its strengths, CPET provides only indirect information on cardiac function during exercise, as central hemodynamic performance is largely inferred from surrogate parameters.

Cardiovascular magnetic resonance imaging (CMR) represents the reference standard for the assessment of cardiac morphology and function, enabling highly accurate and reproducible quantification of ventricular volumes, stroke volume, and cardiac output (6). Recent advances in cardiac real-time magnetic resonance imaging (RT-MRI) have enabled continuous free-breathing (FB) imaging with high temporal resolution, thereby allowing the assessment of cardiac function under physiological conditions and physical exercise (7–10). Previous studies have highlighted the importance of evaluating cardiac performance under physiological conditions, particularly during FB, and have demonstrated the potential of RT-MRI to identify novel diagnostic parameters, including the non-invasive characterization of the Frank-Starling relationship (7, 10–13). The combination of RT-MRI with physical exercise enables highly accurate, non-invasive quantification of cardiac function during stress (14). However, exercise CMR lacks direct information on metabolic demand, ventilatory efficiency, and gas exchange, and therefore cannot assess whether a given hemodynamic response is appropriate for the achieved level of exertion.

The combination of both modalities, commonly referred to as MR-CPET, enables simultaneous measurement of VO₂ and CMR-derived cardiac output during exercise (15). This allows a non-invasive, Fick-based assessment of oxygen transport by combining measured VO₂ with imaging-derived cardiac output (16). In the current implementation, the arteriovenous oxygen difference (a-vO₂ diff) is calculated according to the Fick equation using an assumed arterial oxygen content rather than direct invasive measurements of arterial and mixed venous oxygen content. Despite this limitation, the derived a-vO₂ diff provides a physiological estimate of peripheral oxygen extraction during exercise. This integrative approach provides a unique mechanistic framework to differentiate whether reduced exercise capacity is predominantly associated with impaired cardiac output (central limitation), impaired peripheral oxygen extraction (peripheral limitation), or a combination of both an insight that neither CPET nor exercise CMR can provide in isolation (15).

Initial studies have already demonstrated the feasibility in healthy volunteers and added value of MR-CPET across different patient populations (17–20).

Despite these advances, existing MR-CPET studies are limited and have predominantly focused on demonstrating feasibility and proof-of-concept, often relying on technically complex or resource-intensive protocols. Standardized and easily applicable protocols remain rare, and normative data in healthy individuals are limited. Therefore, the aim of this study was to develop a practical and easy-to-use MR-CPET protocol by implementing user-friendly postprocessing software and an initial characterization of physiological responses in healthy subjects, providing a basis for future large-scale investigations aimed at establishing reference values and future applications in patients with heart failure or impaired cardiac reserve.

## Material and methods

### Study population

This prospective, observational, *in vivo* RT-MRI study investigated healthy, adult volunteers (*n* = 12) and was conducted at the University Hospital Düsseldorf (Heinrich Heine University, Düsseldorf, Germany). All participants provided written informed consent prior to study participation. The study protocol was approved by the local ethics committee (Ethics Committee of the Medical Faculty, Heinrich Heine University Düsseldorf, Germany; approval number 2021-1731) and conducted in accordance with the principles of the Declaration of Helsinki.

### CPET

**Figure 1:**
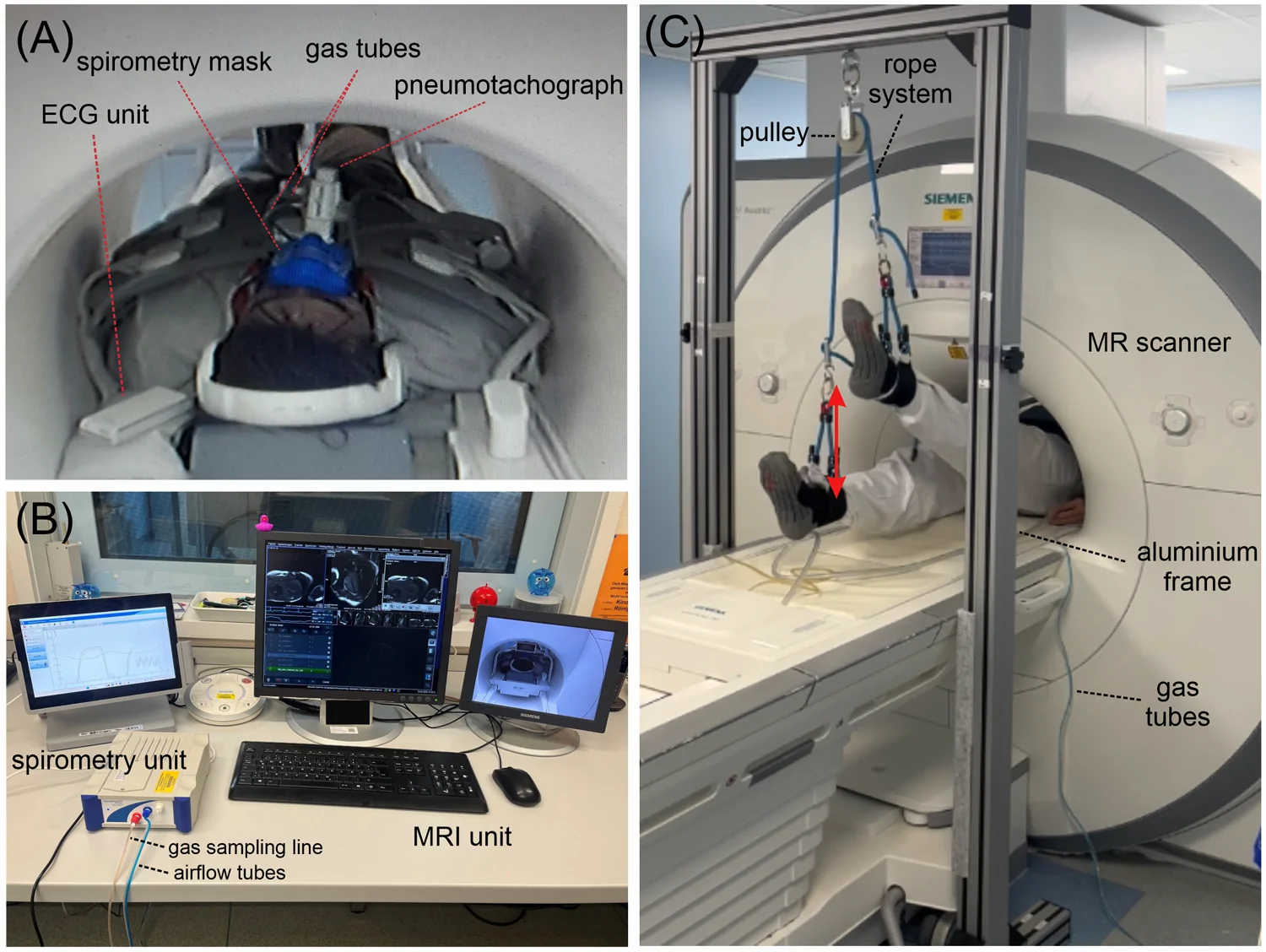
Experimental setup for combined cardiopulmonary exercise testing and RT-MRI. **(A)** Volunteer positioned inside the MR scanner wearing a silicone face mask (COSMED Deutschland GmbH, Werneck, Germany; size M) connected to a non-magnetic lightweight flow sensor enabling continuous measurement of respiratory airflow and gas exchange under MR conditions. The ECG unit was positioned close to the participant's head to minimize motion-induced artifacts. **(B)** Spirometry unit and MRI unit in the control room. The airflow sensor and gas sampling interface were connected to the spirometry unit located in the MR control room via a 10 m airflow tube and a 10 m gas sampling line. **(C)** Exercise was performed in the supine position using an MR-compatible exercise setup. The participant's feet were secured to a rope system routed over a pulley attached to a custom-designed aluminum frame, allowing cyclic up-and-down leg movements to generate submaximal workload inside the MR scanner. ECG, electrocardiogram; MR, magnetic resonance; MRI, magnetic resonance imaging; RT-MRI, real-time magnetic resonance imaging.

CPET was performed using an MR-compatible spirometry (Geratherm Respiratory GmbH, Bad Kissingen, Germany) enabling continuous measurement of respiratory airflow and gas exchange under MR conditions. To allow acquisition within the MRI environment, the participants were wearing a silicone face mask (COSMED Deutschland GmbH, Werneck, Germany, size S or M) that was connected to a non-magnetic light-weight flow sensor (Figure 1A). The airflow sensor and gas sampling interface were connected to the technical spirometry unit located in the MR control room via a 10 m airflow tube and a 10 m gas sampling line, respectively, as previously described (Figure 1B) (12, 21). Prior validation studies demonstrated that the extended tubing did not significantly affect airflow measurements or gas concentration despite the increased dead space even during increased flow (see Supplementary Data Sheet 1 Part A). Respiratory airflow and gas exchange parameters were continuously recorded during CMR acquisitions at rest and during exercise. Submaximal exercise was performed in supine position inside the MR scanner, using a previously described slightly modified MR-compatible exercise setup [for details see video: Submaximal Physical Exercise in MRI - Deutsches Herzzentrum München (22, 23) (Figure 1C)]. Thereby, participant's feet were secured to a rope system routed over a pulley attached to a custom-designed aluminum frame. The system provides an intrinsically auto-normalizing workload, with resistance determined by the participant's body length and body weight (Figure 1C) (23).

### Physiological monitoring

ECG and respiratory bellows signal were recorded using the Siemens Physio logging (VE11C). The ECG physiological reference unit (PERU) was placed at head level to avoid motion artifacts in the ECG signal. Blood pressure and arterial oxygen saturation could be measured at rest and during exercise using the MR-compatible patient monitor Expression MR 200 (Philips, Hamburg, Germany).

### CMR protocol

#### CMR at rest

CMR was performed on a clinical 1.5 T MR scanner (MAGNETOM Avanto fit, Siemens Healthineers, Erlangen, Germany; software version syngo MR E11) with a 32-channel spine matrix coil (direct connect spine 32) and an 18-channel body coil (Body 18, both Siemens Healthineers, Erlangen, Germany).

A standard CMR protocol was applied, including cardiac localizers and electrocardiogram (ECG)-gated balanced steady-state free precession cine sequences to acquire two-, three-, and four-chamber views and left ventricular outflow tract cine images during end-expiratory breath-holding (BH). Subsequently, left ventricular volumetric cine stacks in short-axis orientation were acquired using a conventional ECG-gated cine sequence during end-expiratory BH and a RT-MRI sequence during FB (Table 1). Following volumetric imaging, aortic flow measurements were obtained at the level of the sinotubular junction of the ascending aorta using a conventional phase-contrast MR sequence during BH (image matrix: 256 pixel, slice thickness: 6 mm; field of view: 320mm × 320 mm, repetition time: 37.76 ms, echo time: 2.44 ms, flip angle: 30°, velocity encoding: 200 cm/s, retrospective ECG-gating) and a phase-contrast RT-MRI sequence during FB (image matrix: 212 pixel, slice thickness: 6 mm, field of view: 320mm × 320 mm, repetition time: 3.33 ms; echo time: 2.14 ms, flip angle: 12°, velocity encoding: 200 cm/s, continuous imaging with 30 frames per second).

**Table 1:** Detailed sequence parameters for volumetric assessment.

sequence parameters	cine MRI	RT-MRI
sequence type	2D b-SSFP	2D b-SSFP
TR/TE (ms)	58.3/ 1.1	3.7/ 1.85
FOV (mm)	360 × 360	360 × 360
image matrix (pixels)	192	200
pixel size (mm/pixel)	1.9 × 1.9 × 8.0	1.8 × 1.8 × 8.0
slices (n)	13–18	13–18
slice thickness (mm)	8	8
interslice gap (mm)	0	0
phases	25	500
orientation	short axis	short axis
flip angle (°)	80	60
bandwidth (Hz/Px)	930	760
ECG synchronization	retrospective	-

b-SSFP, balanced steady-state free precession; ECG, electrocardiography; FOV, field of view; TE, echo time; TR, repetition time.

#### CMR during exercise

Prior to the CMR measurements, all participants completed a standardized five-minute warm-up phase. The legs were alternately raised and lowered at a rate of 72 cycles per minute per leg. To implement this, the participants listened to music with a corresponding beat that set the tempo. Subsequently, exercise was continued at the same cadence while RT-MRI acquisitions were performed, including short-axis volumetric imaging and phase-contrast flow measurements in the ascending aorta. RT-MRI during exercise was maintained for approximately 4 min. This results in a total continuous exercise time of 9 min (5 min warm-up, 4 min exercise RT-MRI).

### Data processing and analysis

The data processing pipeline was developed to automate the evaluation of MR-compatible spirometry and to prepare cardiac RT-MRI data for subsequent analysis. The workflow consisted of three primary stages: (1) automated evaluation of spirometry data, including respiratory flow, respiratory volume, and respiratory gas exchange, separately for rest and exercise phases; (2) temporal synchronization of MR-compatible spirometry channels with RT-MRI image data and physiological signals (electrocardiogram and respiratory bellows); and (3) respiratory- and ECG-based binning of RT-MRI data to reconstruct short-axis image stacks with 25 cardiac phases per slice during end-expiration. Processing of RT-MRI and MR-compatible spirometry data was performed using Python (version 3.13.5). The source code and a detailed description of the processing pipeline are publicly available online: https://github.com/MPR-UKD/Realtime-MRI-Pipeline.

Following RT-MRI data processing, the short-axis function module and the 2D flow module of the commercially available post-processing software cvi42 [release 5.17.1 (3504); Circle Cardiovascular Imaging Inc., Calgary, Canada] were used for volumetric and flow analyses. Regions of interest (ROIs) for aortic flow quantification as well as left ventricular endocardial and epicardial contours were generated automatically. Minimal and maximal aortic flow values were automatically identified by the software based on the mean of 2 × 2-pixel ROIs. Whenever required, manual corrections were performed by a medical physician with five years of experience in cardiovascular magnetic resonance imaging (L.R.), applying a standardized approach in accordance with current recommendations for CMR analysis (24, 25).

### Assessment of oxygen consumption and cardiac index according to the Fick principle

VO₂i was directly measured by respiratory gas analysis, whereas CI was obtained from RT-MRI. The a-vO_2_ diff was calculated using the Fick equation (16).

Arterial oxygen content was assumed to be 20 mL/dL, consistent with physiological values in healthy individuals with normal hemoglobin levels and high arterial oxygen saturation (SpO_2_ ≥ 97%) as confirmed by pulse oximetry. Mixed venous oxygen content was calculated as CₐO_2_ minus a-vO_2_ diff. Oxygen extraction fraction was calculated as the ratio of a-vO_2_ diff to arterial oxygen content.

### Reproducibility

The assessment of LV volumes was repeated by the initial rater (L.R.) and a second rater (F.P.) to assess intra- and interrater reliability. Data processing including automate evaluation of MR-compatible spirometry preparation of cardiac RT-MRI data for subsequent analysis was repeated in 3 subjects to assess processing time reproducibility.

### Comfort Survey

After the measurements, all volunteers completed a standardized questionnaire assessing their comfort during the examination. Various aspects of the study protocol were evaluated, including overall comfort and examination duration, comfort during the resting phase, comfort during the exercise phase, duration of the exercise phase, as well as comfort related to the spirometry mask, the MR-compatible ergometer, ECG and blood pressure measurement. All items were rated on a 5-point Likert scale ranging from 1 (very comfortable) to 5 (extremely unpleasant).

### Statistical Analysis

Statistical analyses were performed using IBM SPSS Statistics for Windows (version 29.0.2.0; IBM Corp., Armonk, NY, USA). Normality of data distribution was assessed using the Shapiro–Wilk test. Continuous variables are reported as mean ± standard deviation. For normally distributed variables, paired-samples t-tests were used to compare CPET parameters, cardiac volumes and Fick components between rest and submaximal exercise conditions. To quantify effect magnitude, Hedges’ g for paired samples was calculated and reported alongside *p*-values. As a small-sample bias-corrected standardized effect size, Hedges’ g was interpreted using conventional Cohen benchmarks: 0.2 = small, 0.5 = medium, and 0.8 = large, while acknowledging their context-dependent nature (26, 27).

Independent-samples t-tests were applied to evaluate sex-specific differences in exercise-induced changes of CPET parameters.

Agreement between CMR measurements obtained using conventional cine MRI and RT-MRI was evaluated using Bland-Altman analysis, reporting the mean bias and 95% limits of agreement (28). Linear associations between CPET parameters and cardiac volumes at rest and during submaximal exercise were evaluated using Pearson correlation coefficients. The effect size rho (*r*) was classified as small (0.1–0.3), medium (0.3–0.5) and strong (> 0.5), according to Cohen (29). For ordinal variables, including comfort questionnaire ratings, the Wilcoxon signed-rank test was used.

Intra- and interrater reliability for LV volumetry measurements were assessed using the intraclass correlation coefficient (ICC) based on a two-way random-effects model with absolute agreement. ICC values were interpreted as excellent (>0.90), good (>0.75 to 0.9), moderate (≥ 0.50 to 0.75), or poor (<0.50) (30).

A level of *p* < 0.05 was considered significant.

## Results

### Study population

All healthy adult volunteers (*n* = 12; male = 6, female = 6) completed the combined cardiovascular RT-MRI CPET exercise protocol without difficulty. The volunteers’ age range was 19 to 34, with an average age of 24 ± 4 years, an average body weight of 67 ± 13 kg, and an average body height of 172 ± 9 cm. Body surface area (BSA) was calculated at 1.8 ± 0.2 m^2^ using the DuBois formula and was used to calculate indexed volumes. The participants were in good physical condition and engaged in an average of two to three training sessions per week involving ball sports or endurance training. The body mass index was 23 ± 3 kg/m^2^. None of the volunteers had a history of cardiovascular disease.

### Data processing

All data records were successfully processed with our custom-written pipeline. The development of dedicated software with graphical user interface significantly facilitated and accelerated the workflow. Evaluation of the MR-compatible spirometry data across both rest and exercise phases required approximately 6 min. The RT-MRI data processing took roughly 20 min, comprising 10 min of manual preparation via the graphical user interface and 10 min of automated processing (Intel® Core™ i7-14700 CPU). Subsequently, all RT-MRI data from resting and exercise phases could be analyzed in the commercial evaluation software. Since the contours of the left ventricle and the aorta required further correction in some cases, the evaluation of a single subject took∼25 min.

### MR-compatible spirometry at rest and during exercise

**Figure 2:**
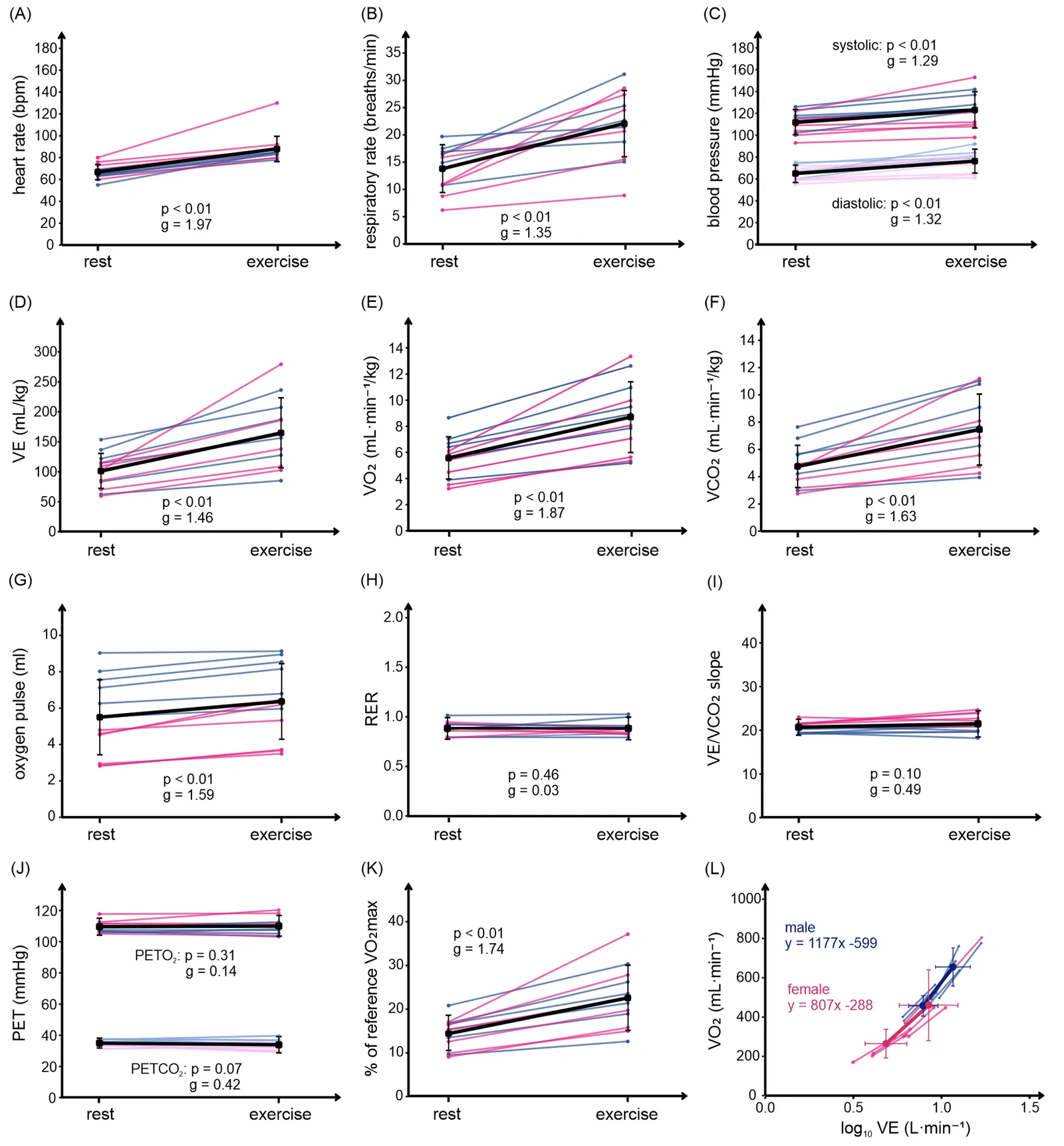
Individual and mean CPET results. **(A–K)** Individual CPET parameters at rest and during exercise for male (blue) and female (pink) volunteers. Mean values with standard deviations are shown in black in. *P*-values from paired-samples t-test are provided. Effect size reported as Hedges’ g. **(L)** Individual and mean OUES values for males and females. For males, individual values are shown as blue lines and dots, and mean values ± standard deviation as dark blue lines with whiskers. For females, individual values are shown as pink lines and dots, and mean values ± standard deviation as dark pink lines with whiskers. bpm, beats per minute; CPET, cardiopulmonary exercise testing; OUES, oxygen uptake efficiency slope; PETCO_2_, end-tidal partial pressure of CO_2;_ PETO_2_, end-tidal partial pressure of O_2_; RER, respiratory exchange ratio; VE, minute ventilation; VCO₂, carbon dioxide output; VO₂, oxygen consumption; VO₂max, maximal oxygen consumption.

Individual and mean CPET parameters at rest and during submaximal exercise are presented in Figures 2A–K. The supine submaximal exercise protocol achieved a mean peak oxygen uptake corresponding to 22.5 ± 6.7% of the age-, sex-, and weight-adjusted predicted reference values reported for healthy individuals during maximal exercise (31) (Figure 2K).

Sex-specific analyses of exercise-induced changes revealed a statistically significant difference only for absolute oxygen uptake efficiency slope (OUES) values. Accordingly, absolute OUES values were analyzed separately for males and females (Figure 2L). No other CPET parameters demonstrated significant sex-related differences in their response to submaximal exercise. Therefore, subsequent analyses of mean values were performed without sex stratification (Figures 2A–K).

During exercise, heart rate, respiratory rate, minute ventilation (VE), blood pressure, oxygen consumption, carbon dioxide output, and oxygen pulse increased significantly compared to resting conditions (Figures 2A–G). In contrast, ventilatory efficiency (VE/VCO₂ slope), respiratory exchange ratio (RER), end-tidal partial pressure of carbon dioxide (PETCO₂), and end-tidal partial pressure of oxygen (PETO₂) did not change significantly between rest and exercise (Figures 2 H–J).

### CMR at rest and during exercise

#### CMR at rest

Conventional cine CMR with BH and cardiac RT-MRI with FB at rest provided good image quality. For details see Supplementary Video 1.

**Figure 3:**
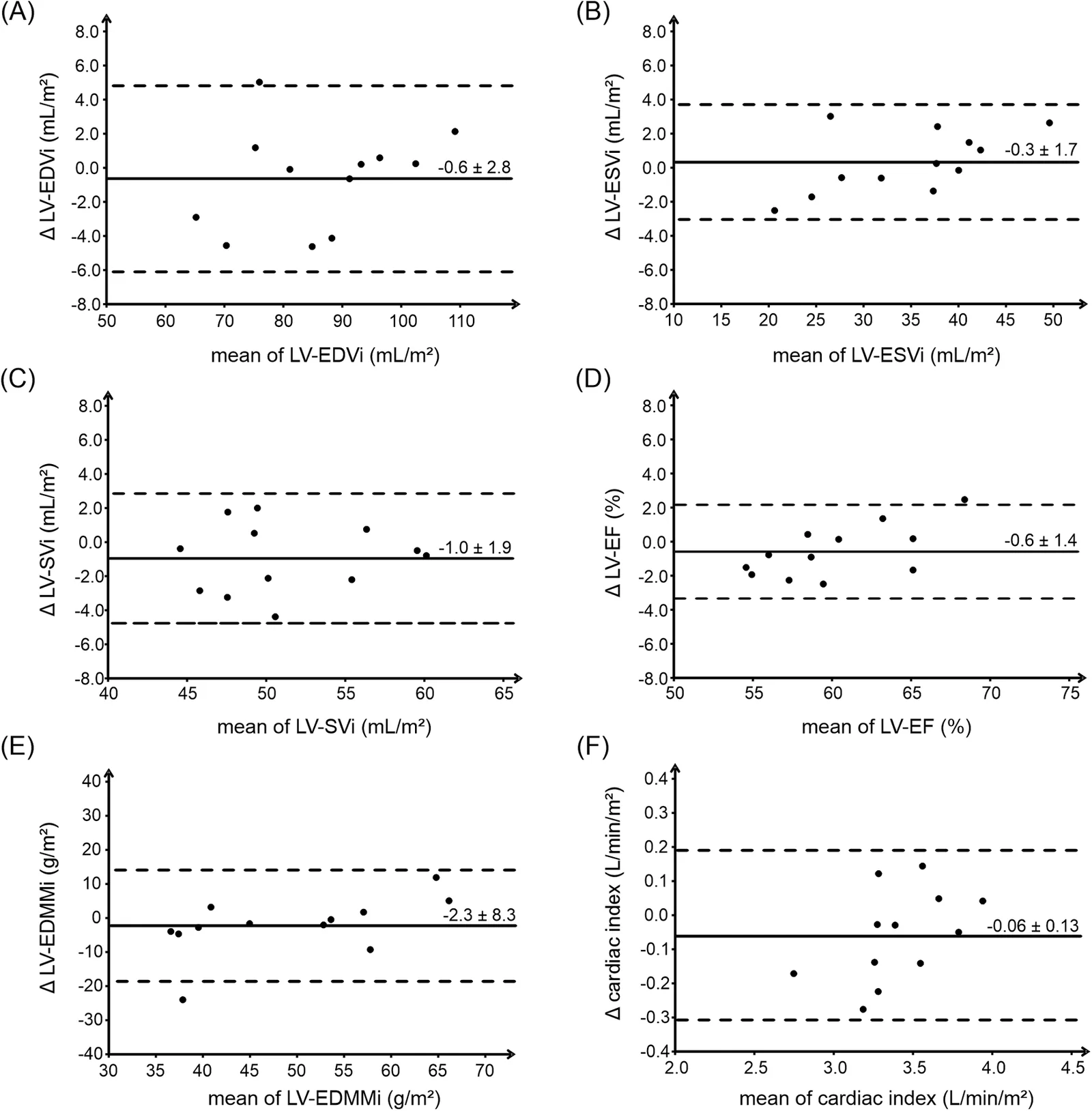
Bland-Altman analysis (conventional cine vs. RT-MRI at Rest). Bland-Altman plots showing cardiac LV volume parameters (left ventricular end-diastolic volume indexed to body surface area **(A)**, left ventricular end-systolic volume indexed to body surface area **(B)**, left ventricular stroke volume indexed to body surface area **(C)** left ventricular ejection fraction **(D)**, left ventricular end-diastolic muscle mass indexed to body surface area **(E)** and cardiac index **(F)** comparing conventional cine CMR and cardiac RT-MRI. *Δ* = difference (RT-MRI vs. conventional cine MRI), solid line = mean value; dashed lines = ± 1.96 standard deviations. Bias +/- Limits of agreement are inserted in the graphs. LV-EDVi, left ventricular end-diastolic volume indexed to body surface area, LV-ESVi, left ventricular end-systolic volume indexed to body surface area, LV-SVi, left ventricular stroke volume indexed to body surface area, LV-EF, left-ventricular ejection fraction, LV-EDMMi, left ventricular end-diastolic muscle mass indexed to body surface area, RT-MRI, real-time magnetic resonance imaging.

Agreement between conventional cine MRI and RT-MRI for LV-EDVi, LV-ESVi, LV-SVi, LV-EF, LV-EDMM_i_, and CI were further assessed using Bland-Altman analysis (Figure 3). The bias and the limits of agreement (LoA) for LV-EDVi were −0.6 (bias) and 2.8 mL/m^2^ (LoA), respectively (Figure 3A). Similar small biases and narrow limits of agreement were observed in the LV-ESVi, LV-SVi and LV-EF (LV-ESVi: bias: −0.3, LoA: 1.7 mL/m^2^, LV-SVi: bias: −1.0, LoA: 1.9 mL/m^2^, LV-EF: bias: −0.6, LoA: 1.4%) (Figures 3B–D), whereas the LV-EDMMi revealed a slightly larger bias and wider limits of agreement (bias: −2.3, LoA: 8.3 g/m^2^) (Figure 3E). The good agreement in LV volumetry resulted in similarly low bias and narrow limits of agreement for the CI (bias: −0.06, LoA: 0.13 L/min/m^2^) (Figure 3F).

Pearson correlation analyses demonstrated strong associations between conventional cine MRI and RT-MRI at rest for left ventricular end-diastolic volume indexed to body surface area (LV-EDVi), left ventricular end-systolic volume indexed to body surface area (LV-ESVi), left ventricular stroke volume indexed to body surface area (LV-SVi), left ventricular ejection fraction (LV-EF), left ventricular end-diastolic myocardial mass indexed to body surface area (LVEDMMi) and cardiac index (CI) (Table 2). Paired-samples t-tests revealed no statistically significant differences between the two imaging modalities for any of these parameters (Table 2).

**Table 2:** Mean left ventricular cardiac volume results and fick-components at rest and during exercise.

variable	cine MRI	RT-MRI		cine vs. RT-MRI at rest	RT-MRI: rest vs. exercise
	rest		exercise		
CMR					
LV-EDVi (mL/m^2^)	86.4 ± 11.8	85.8 ± 12.6	80.1 ± 10.9	*r* = 0.98 *p* = 0.47	*r* = 0.94 *p* = 0.001
LV-ESVi (mL/m^2^)	34.6 ± 7.4	34.9 ± 8.3	31.7 ± 6.9	*r* = 0.98 *p* = 0.55	*r* = 0.96 *p* = 0.002
LV-SVi (mL/m^2^)	51.8 ± 4.8	50.9 ± 5.0	48.4 ± 5.0	*r* = 0.93 *p* = 0.13	*r* = 0.77 *p* = 0.04
LV-EF (%)	60.4 ± 3.7	61.5 ± 7.2	60.8 ± 4.3	*r* = 0.61 *p* = 0.55	*r* = 0.87 *p* = 0.20
LVEDMMi (g/m^2^)	50.2 ± 8.5	48.0 ± 12.7	49.7 ± 10.1	*r* = 0.78 *p* = 0.39	*r* = 0.88 *p* = 0.39
net aortic forward flow (mL/m^2^)	49.5 ± 4.5	46.5 ± 5.4	46.5 ± 5.4	*r* = 0.95 *p* = 0.004	*r* = 0.89 *p* = 0.99
peak velocity aortic flow (m/s)	1.1 ± 0.1	1.2 ± 0.2	1.2 ± 0.2	*r* = 0.90 *p* = 0.003	*r* = 0.65 *p* = 0.60
heart rate (bpm)	66.8 ± 6.4	66.8 ± 6.4	88.1 ± 12.6	*r* = 1.0 -	*r* = 0.72 *p* = 0.00004
cardiac index (L/min/m^2^)	3.4 ± 0.3	3.4 ± 0.3	4.2 ± 0.6	*r* = 0.93 *p* = 0.16	*r* = 0.74 *p* = 0.00001
spirometry					
peak VO_2_i during the protocol (mL/min/m^2^)	-	201.2 ± 55.2	309.1 ± 89.2	-	*r* = 0.86 *p* = 0.00001
a-vO_2_ diff (mL/dL)	-	5.9 ± 1.5	7.2 ± 1.4	-	*r* = 0.90 *p* = 0.0002
O_2_ extraction (%)	-	29.3 ± 7.3	35.9 ± 6.9	-	*r* = 0.90 *p* = 0.0002

Statistics: Pearson correlation coefficient (r), paired-samples t-test (*p*-value).

a-vO_2_ diff, arteriovenous oxygen difference, bpm, beats per minute; LV-EDVi, left ventricular end-diastolic volume indexed to body surface area; LV-EDMMi, left ventricular end-diastolic myocardial mass indexed to body surface area; LV-EF, left ventricular ejection fraction; LV-ESVi, left ventricular end-systolic volume indexed to body surface area; LV-SVi, left ventricular stroke volume indexed to body surface area; RT-MRI, real-time magnetic resonance imaging, VO_2_i, oxygen consumption indexed to body surface area.

For aortic flow measurements, Pearson correlation analyses demonstrated strong associations between conventional cine MRI and RT-MRI for net aortic forward flow and peak aortic flow velocity (Table 2). However, paired-samples t-tests indicated statistically significant differences between the two imaging modalities for these parameters (Table 2).

#### Cardiac RT-MRI during exercise

Mild thoracic motion occurred during exercise; however, RT-MR image quality was not substantially affected. Endocardial and epicardial delineation of the left ventricle remained adequate for analysis throughout submaximal exercise. For details see Supplemental Video 1.

**Figure 4:**
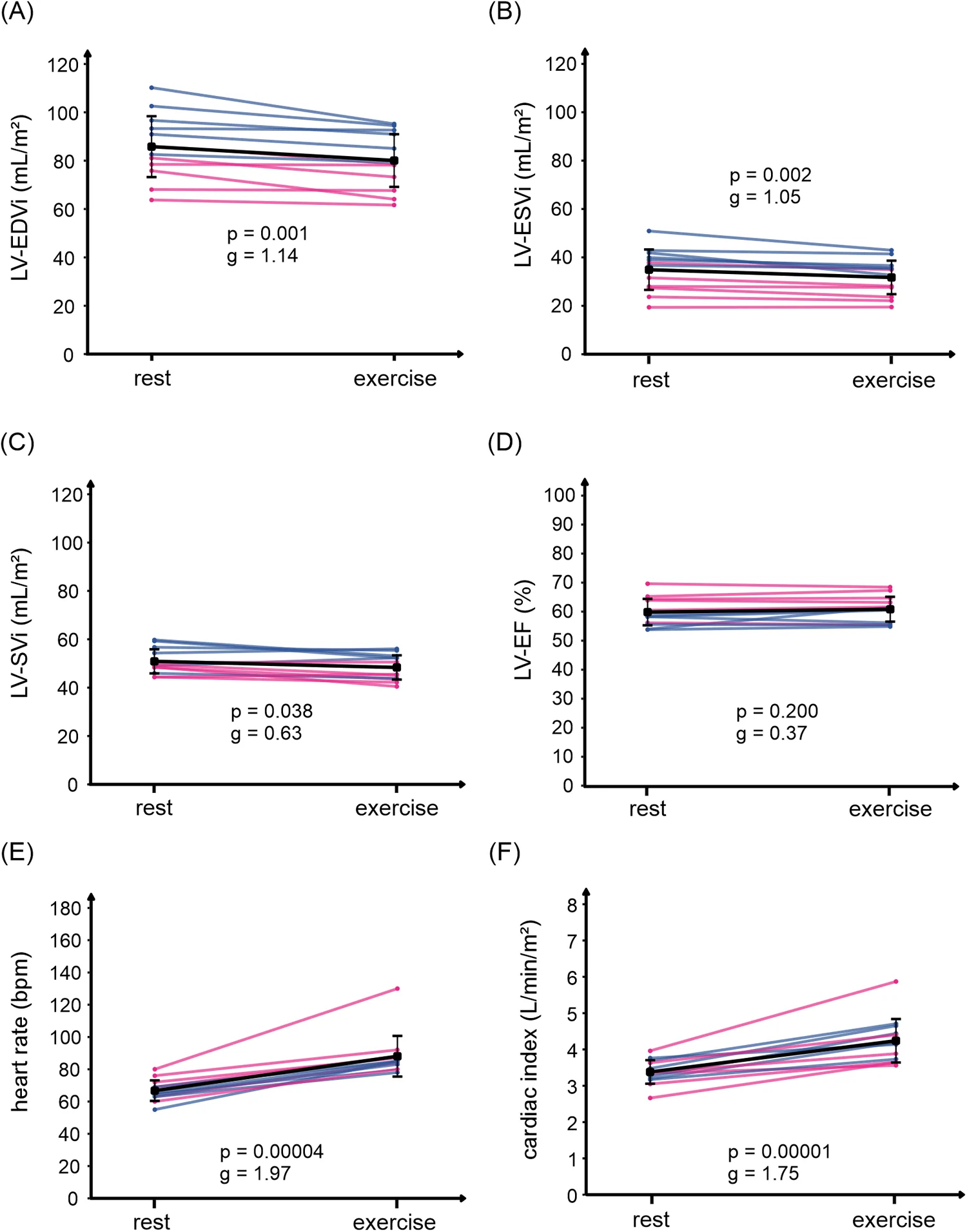
Individual and mean cardiac volumes at rest and during exercise. Individual and mean cardiac volumetric parameters (left ventricular end-diastolic volume **(A)**, end-systolic volume **(B)**, stroke volume **(C)**, and ejection fraction **(D)**) indexed to body surface area obtained during RT-MRI at rest and during submaximal exercise. Data are shown separately for male (blue lines and dots) and female volunteers (pink lines and dots). Mean values ± standard deviations are displayed in black (lines with whiskers). *P*-values from paired-samples t-test are provided. Effect size reported as Hedges’ g. LV-EDVi, left ventricular end-diastolic volume indexed to body surface area, LV-ESVI, left ventricular end-systolic volume indexed to body surface area, LV-SVi, left ventricular stroke volume indexed to body surface area, LV-EF, left ventricular ejection fraction; RT-MRI, real-time magnetic resonance imaging.

During exercise, LV-EDVi, LV-ESVi, and LV-SVi showed a small but statistically significant decrease (Figures 4A–C, Table 2). In contrast, LV-EF and LV-EDMMi did not change significantly and remained stable under exercise conditions (Figure 4D, Table 2). The increase in the cardiac index is caused by an increase in heart rate during exercise (Figures 4E,F, Table 2). Aortic flow measurements demonstrated no significant differences in net forward flow or peak flow velocity between rest and submaximal exercise (Table 2).

#### Fick-derived changes in oxygen consumption and cardiac index during exercise

**Figure 5:**
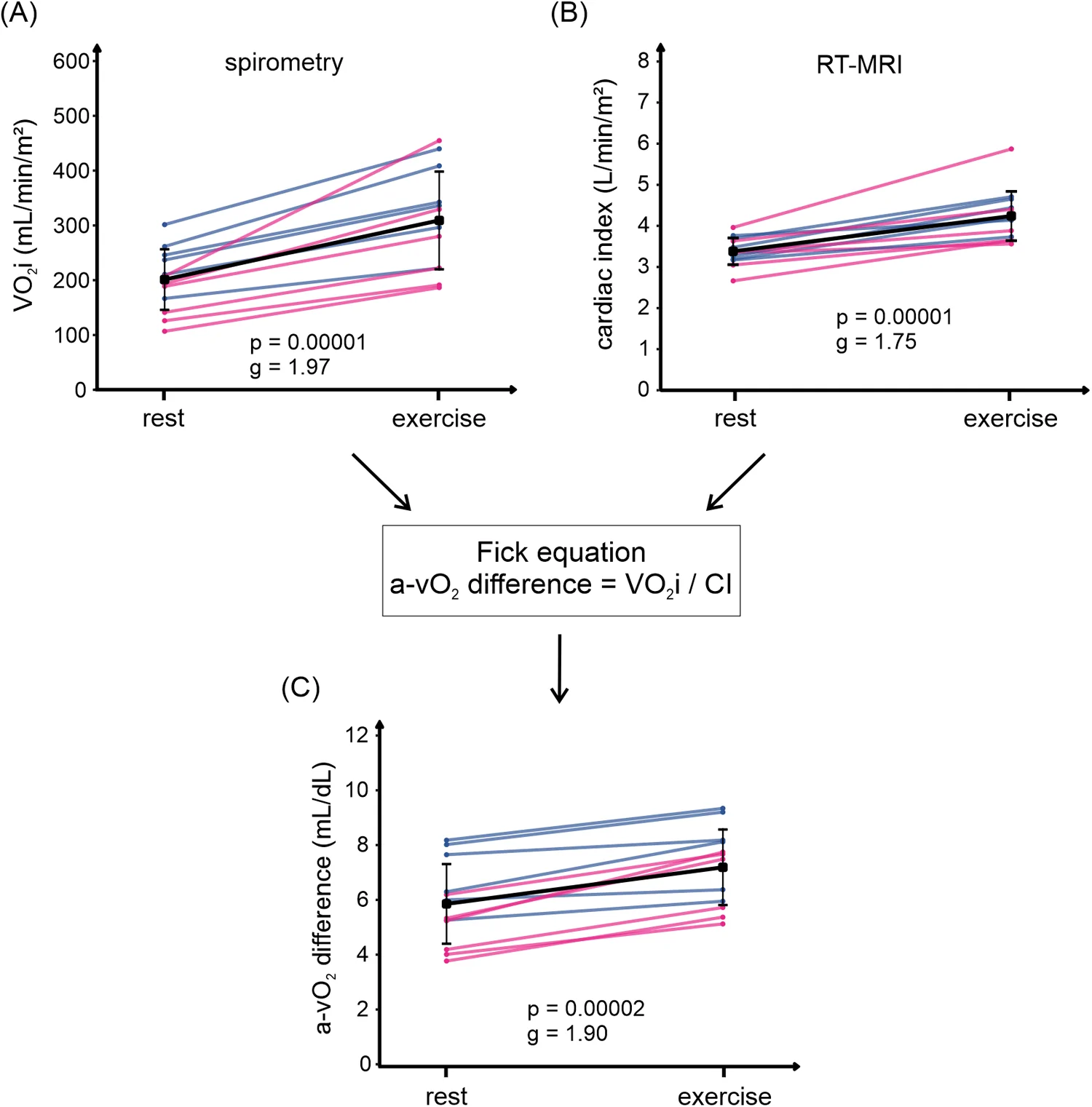
Fick-derived parameters at rest and during exercise. Fick parameters including VO2i derived from spirometry **(A)**, cardiac index derived from RT-MRI **(B)**, a-vO_2_ diff calculated according to the Fick principle **(C)** at rest and during exercise. Data are presented separately for male (blue lines and dots) and female volunteers (pink lines and dots). *P*-values from paired-samples t-test are provided. Effect size reported as Hedges’ g. a-vO_2_ diff, arteriovenous oxygen difference; CI, cardiac index; O_2_, oxygen; RT-MRI, real-time magnetic resonance imaging; VO_2_i, oxygen consumption indexed to body surface area.

At rest, indexed oxygen consumption was 201 ± 55 mL/min/m^2^ with a cardiac index of 3.4 ± 0.3 L/min/m^2^ (Table 2, Figures 5A,B). During exercise, VO₂i increased to 309 ± 89 mL/min/m^2^, accompanied by an increase in cardiac index to 4.2 ± 0.6 L/min/m^2^ primarily induced by an increase in the heart rate (Table 2, Figures 5A,B). According to the Fick relationship, the calculated a-vO_2_ diff increased from 5.9 ± 1.5 mL/dL at rest to 7.3 ± 1.4 mL/dL during exercise (Figure 5C). Assuming an arterial oxygen content of approximately 20 mL/dL (32), the corresponding mixed venous oxygen content decreased from 14.1 ± 1.5 mL/dL to 12.8 ± 1.4 mL/dL. These values correspond to an oxygen extraction fraction of approximately 30 ± 7% at rest and 36 ± 7% during exercise. Paired-samples t-test proved the differences of the Fick components at rest and during exercise were statistically significant (Figures 5A–C, Table 2).

#### Reproducibility

The ICC was good to excellent for the intra- and interrater analysis of the conventional LV volumetry as well as for RT-MR LV volumetry at rest and during exercise. For details see: Supplementary Data Sheet 1 Part B: Supporting Table S1. The reevaluation of processing time in three subjects yielded similar results to the initial evaluation, with the analysis of MR-compatible spirometry taking approximately 5 min and the preparation of RT-MRI data taking about 25 min (approximately 15 min of manual data entry into the analysis tools and 10 min of automated processing).

#### Comfort survey

**Figure 6:**
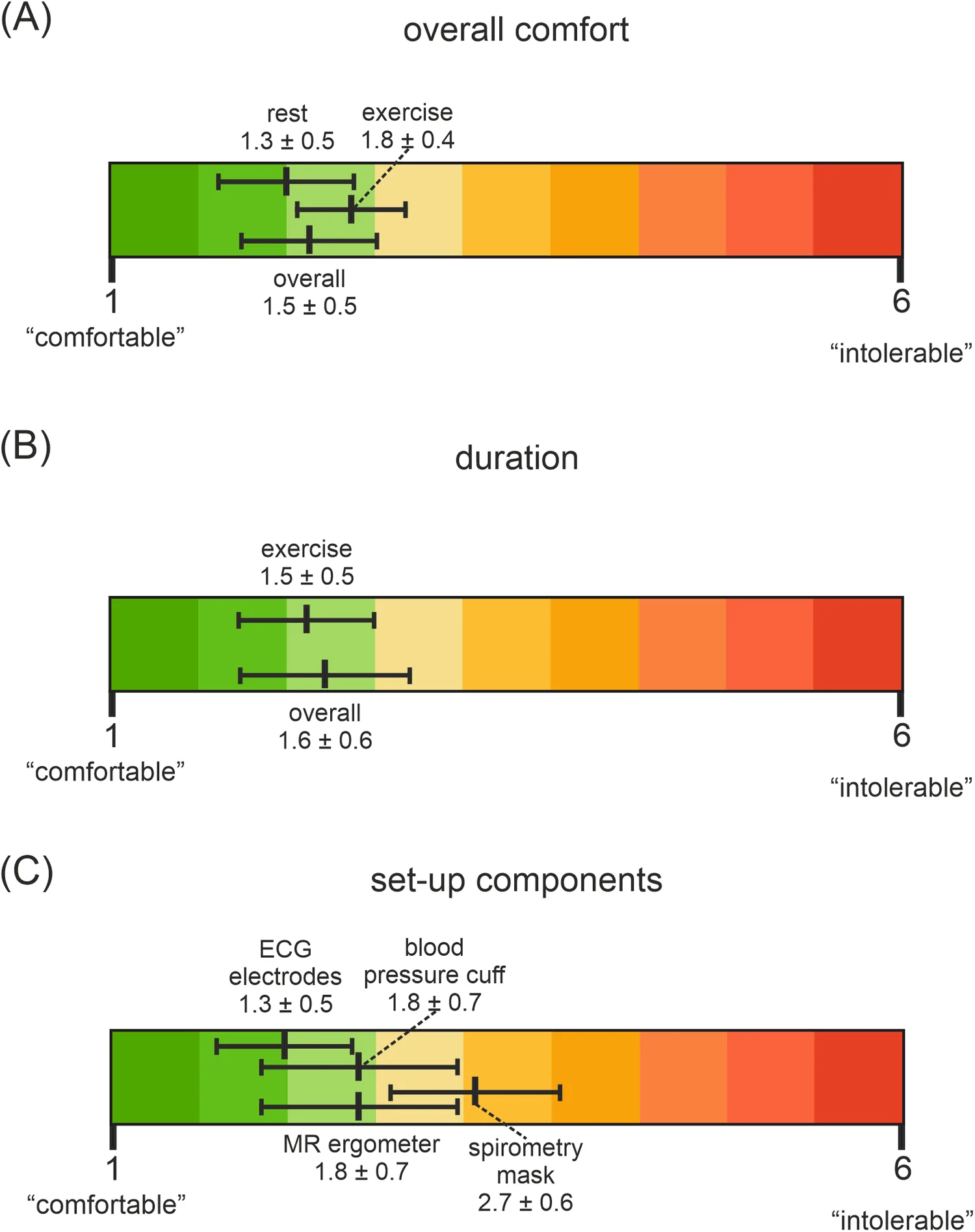
Participant-Reported comfort during combined CPET and RT-MRI. Mean comfort ratings for the overall examination and for RT-MRI performed at rest and during supine exercise **(A)** Perceived duration of the entire examination and of the RT-MRI exercise phase **(B)** Comfort ratings for individual setup components, including ECG electrodes, blood pressure cuff, MR-compatible ergometer, and spirometry mask **(C)** Data are presented as mean ± standard deviation. CPET, cardiopulmonary exercise testing; ECG, electrocardiogram; MR, magnetic resonance; RT-MRI, real-time magnetic resonance imaging.

The results of the comfort survey indicate that the examination was generally well tolerated (Figure 6A). Although comfort ratings were lower during stress compared with resting conditions, the procedure was still predominantly described as comfortable (Figure 6A). Both the overall examination time and the duration of the stress phase were rated as acceptable (Figure 6B). Among the individual components, only the spirometry mask was perceived as slightly uncomfortable, whereas the MR-ergometer, blood pressure cuff, and ECG monitoring were reported to have lower impact on overall comfort (Figure 6C).

## Discussion

The main findings of this study are the following: First, the combined CPET and RT-MRI setup proved feasible and was well tolerated during submaximal supine exercise. Second, volumetric parameters derived from RT-MRI during FB using combined ECG and respiratory-based binning showed good agreement with conventional cine imaging during BH at rest, supporting the validity of the imaging approach. Third, the integrated assessment of metabolic and hemodynamic parameters revealed physiologically consistent increases in oxygen uptake, CI, and a-vO_2_ diff during exercise.

The results of the comfort survey indicate overall good tolerability of the experimental setup, with only a minor reduction in comfort attributable to the spirometry mask. All participants were able to complete the exercise protocol without difficulty. The protocol was deliberately limited to submaximal workloads reflecting activities of daily living, that should be more suitable for patient populations in whom maximal exercise is often limited or contraindicated (33, 34). Although commercially available MR-compatible ergometer systems permit higher, including near-maximal, exercise intensities (15), we intentionally used a simple, broadly applicable protocol requiring relatively inexpensive equipment to facilitate future clinical translation, particularly in patients with impaired exercise capacity. Despite the submaximal workload, the protocol elicited consistent increases in heart rate, CI, VO₂, and Fick-derived a-vO₂ diff, demonstrating measurable adaptations in both central and peripheral components of oxygen transport. Previous exercise CMR studies have likewise shown that submaximal exercise provides clinically relevant physiological information, including improved assessment of ventricular function and valvular performance in congenital heart disease (22) and dynamic changes in aortic regurgitation not evident at rest (23). Although higher exercise intensities may reveal abnormalities not detected during submaximal exercise, submaximal workloads are often sufficient to unmask clinically meaningful cardiovascular dysfunction while providing more stable imaging conditions and longer acquisition times (22, 23, 35, 36). Future studies may combine the proposed post-processing workflow with commercially available MR-compatible ergometer systems to investigate higher exercise intensities where appropriate, for example in physiological studies of healthy participants.

The good agreement between RT-MRI and conventional cine MRI at rest confirms the reliability of RT-MRI for the assessment of ventricular volumes and function. This is relevant, as motion and breathing preclude conventional cine MRI during exercise. The present findings indicate that RT imaging combined with respiratory and ECG-based binning allows quantification of cardiac function under dynamic conditions. This is in line with previous studies that combine fast imaging techniques e.g., RT-MRI or compressed sensing during supine exercise (37, 38). In prior exercise RT-MRI studies, respiratory binning has been performed using different approaches including image-derived respiratory surrogates (39, 40), and plethysmography-based respiratory phase definition (7, 41). The present study introduces respiratory flow and tidal volume data obtained from MR-compatible spirometry for respiratory-based binning of RT-MRI. This approach extends existing methodologies and should facilitate future investigations of heart-lung interactions during exercise.

At rest, participants exhibited LV-EDVi and LV-SVi in the upper range of sex-specific reference values (42). This is probably explained by the increase of venous return and preload due to supine position with leg elevation, leading to enhanced stroke volume via the Frank-Starling mechanism (43, 44). Accordingly, CI values were already elevated at rest. The observed increase in CI during exercise was predominantly driven by heart rate, which is consistent with known physiological responses under supine conditions, where stroke volume reserve may be partially limited due to already elevated preload (45). Consequently, a greater reliance on chronotropic mechanisms is required. This observation is in line with previous supine exercise MRI studies (14, 22, 46).

The present findings further demonstrate a physiologically consistent relationship between VO_2_, and cardiac output as described by the Fick principle (16, 47). During moderate exercise, the increase in VO_2_i was accompanied by a proportional rise in CI as well as a moderate increase in the calculated a-vO_2_ diff. This pattern reflects the expected integrative cardiopulmonary response to submaximal exercise, where oxygen delivery is augmented by both central (cardiac output) and peripheral (tissue oxygen extraction) mechanisms. The calculated oxygen extraction fraction increased from approximately 30% at rest to 36% during moderate exercise, which lies within the expected physiological range for healthy individuals (48, 49). While these values are derived and not directly measured, their consistency with established physiological ranges supports the plausibility of the approach.

The observed increase in CI together with the corresponding increase in Fick-derived a-vO2 diff is consistent with coordinated physiological adaptation of oxygen delivery and utilization during submaximal exercise. However, as oxygen extraction was derived indirectly according to the Fick principle, these findings should be interpreted as physiological estimates rather than direct measurements.

In this study, we developed and validated an easy-to-use experimental setup that integrates MR-compatible spirometry with cardiac RT-MRI during supine exercise. This approach enables the simultaneous assessment of oxygen uptake, cardiac output, and Fick-derived parameters, providing a comprehensive evaluation of cardiopulmonary function. The developed postprocessing software facilitates a user-friendly workflow that can be performed within a reasonable time frame. Fully integrated protocols combining continuous gas exchange measurements with simultaneous cardiac imaging have not yet been widely established. Even with the streamlined postprocessing approach presented in our study, implementing CPET remains challenging because radiology technologists are generally unfamiliar with the procedure.

While MR-CPET typically yields a lower VO₂max than conventional upright CPET (50, 51) and does not achieve the image quality of conventional CMR, the simultaneous acquisition of gas exchange and CMR during exercise provides important complementary information. Conventional CPET provides a comprehensive assessment of exercise performance, and treadmill-based protocols are well suited for determining VO₂max. In contrast, MR-CPET enables quantification of the relative contributions of cardiac output and peripheral oxygen extraction to oxygen uptake while providing a mechanistic understanding of exercise intolerance. By integrating measurements of VO₂ with imaging-derived cardiac output and Fick-derived estimation of a-vO₂ diff obtained under submaximal exercise conditions, MR-CPET enables assessment of these determinants. This integrated approach may improve mechanistic understanding of exercise intolerance beyond surrogate measures obtained from separate examinations and may therefore provide additional diagnostic value in patients with unexplained exercise intolerance or complex cardiopulmonary disease.

The present study focused on LV function because the primary objective was to establish a feasible MR-CPET workflow and to evaluate Fick-derived measures based on systemic cardiac output. Nevertheless, the acquired RT-MRI data and post-processing approach are not inherently limited to left ventricular assessment. Future studies in patient populations with pulmonary vascular disease or congenital heart disease may benefit from incorporating dedicated right ventricular imaging, enabling comprehensive biventricular evaluation and assessment of cardiopulmonary interactions during exercise.

Beyond this proof-of-concept study, the integrated MR-CPET workflow presented here provides a practical technical foundation for future translational research. By combining the simultaneous assessment of cardiac function, pulmonary gas exchange, and peripheral oxygen utilization, MR-CPET has the potential to improve the mechanistic phenotyping of patients with unexplained exercise intolerance or complex cardiovascular diseases. Rather than replacing conventional CPET or CMR, its future role is likely to be complementary, providing additional physiological information where conventional diagnostic tools remain inconclusive. However, further clinical studies are needed to demonstrate its diagnostic value before routine clinical use can be recommended.

Previous studies in patient populations have demonstrated the diagnostic value of MR-CPET especially in clinical populations, where distinguishing between central and peripheral limitations to exercise capacity is essential (17, 18, 20, 52). For example, impaired oxygen extraction has been identified as the dominant limitation in systemic sclerosis (20), whereas reduced cardiac output plays a larger role in pulmonary hypertension phenotypes (17). Similarly, in cancer survivors with persistent fatigue, reduced exercise capacity was primarily attributed to impaired cardiac reserve rather than peripheral extraction (18). The presented workflow therefore might provide a valuable tool for future clinical studies investigating patients with heart failure, pulmonary hypertension, cardio-oncological conditions or unexplained exercise intolerance. By enabling simultaneous assessment of exercise physiology and cardiac function, MR-CPET has the potential to improve characterizing the pathomechanism of exercise limitation and may support more individualized diagnostic evaluation in selected patient populations. However, the present study was conducted exclusively in healthy volunteers and was designed as a technical feasibility study. Consequently, the current findings do not permit conclusions regarding diagnostic performance or clinical decision-making. Prospective studies in larger and disease-specific cohorts are required to establish reproducibility, diagnostic accuracy, incremental clinical value, and the impact of MR-CPET on patient management before broader clinical implementation can be recommended.

### Limitations

Several limitations of this feasibility study should be acknowledged when interpreting the present findings and considering their potential clinical applicability. The study was conducted in a small cohort of healthy volunteers (*n* = 12), which limits the generalizability of the findings to clinical populations and permits only preliminary physiological observations in healthy subjects. Larger cohorts are required to establish reference values in a healthy population. Furthermore, because only healthy volunteers were included, the present study cannot determine the diagnostic performance of MR-CPET or its ability to differentiate between specific pathophysiological mechanisms in patients with cardiovascular disease.

Furthermore, exercise intensity in the supine position was restricted to submaximal levels due to technical constraints of the MR-ergometer. Exercise performance is also inherently influenced by subject motivation, and standardization between individuals remains limited.

Although volumetric parameters derived from RT-MRI showed good agreement with conventional cine MRI, image quality was slightly reduced compared to standard cine acquisitions. This required additional time-consuming manual correction of left ventricular and aortic contours. An additional consideration relates to the assumption of a fixed arterial oxygen content of 20 mL/dL for calculation of the a-vO₂. Because calculating arterial oxygen content from pulse oximetry requires knowledge of the hemoglobin concentration, our assumed hemoglobin value introduced uncertainty into the absolute a-vO₂ difference values in our study. Normal hemoglobin concentrations in healthy adults are typically confined to a physiological range of approximately 12–16 g/dL in women and 13–18 g/dL in men (53), corresponding to arterial oxygen contents of approximately 17–22 mL/dL under normal oxygen saturation conditions. Therefore, this assumption may have influenced the observed exercise-induced trends in oxygen extraction. Future clinical MR-CPET studies therefore should aim to include a same-day hemoglobin measurement, which is routinely obtained in many patient populations and could be coordinated with the imaging examination.

While the experimental setup was generally well tolerated, minor discomfort was reported, mainly related to the spirometry mask. Finally, despite optimization of post-processing through automated CPET analysis, automated synchronization of MR-compatible spirometry with RT-MRI, and respiratory- and ECG-based binning, data analysis remains time-consuming, thereby limiting its applicability in routine clinical practice.

## Conclusion

Combined CPET and RT-MRI using the presented, user- and participant- friendly setup enables an integrated assessment of cardiopulmonary performance and cardiovascular function during submaximal exercise. This approach provides physiologically plausible Fick-derived measures of oxygen transport and utilization. The inclusion of advanced postprocessing tools, including respiratory binning based on MR-compatible spirometry, further facilitates data analysis and enables its application as a promising tool for investigating heart-lung interactions. This setup may have clinical value in future studies by helping distinguish central from peripheral limitations to exercise capacity.

## Data Availability

The original contributions presented in the study are included in the article/Supplementary Material, further inquiries can be directed to the corresponding author.

## Supplementary material

Supplementary Data Sheet 1 Part A: MR-compatible spirometry: Validation of extended-length (10 m) tubes. Part B: Results of Reproducibility Analysis of Left Ventricular Cardiac Volumes

Supplementary Video 1 Images of a midventricular slice from a representative volunteer acquired during cardiac conventional cine MRI during breath-holding (A), cardiac real-time MRI during free-breathing at rest (B) and during submaximal exercise (C). BH = breath-holding, FB = free-breathing, MRI = magnetic resonance imaging, RT-MRI = real-time magnetic resonance imaging.
