# Supplementary material for: Real-time CMR combined with cardiopulmonary exercise testing enables integrated assessment of cardiac function during exercise
Source: Front Cardiovasc Med. 2026 Aug 11;13:1873004. doi: 10.3389/fcvm.2026.1873004 (PMC13503221; doi:10.3389/fcvm.2026.1873004)
Supplement: Supplementary file 1 [file Datasheet1.pdf]

Supporting Information

Part A)

MR-compatible spirometry: Validation of extended-length (10 m) tubes.

Background: The aim of the validation study was to determine whether the longer tube required to perform spiroergometry within the MRI, and the resulting increase in dead space, has an effect on flow curves and respiratory gas measurements (oxygen, carbon dioxide).

Material and Methods: A standardized setup with a metabolic simulator (VacuMed Metabolic Simulator, https://vacumed.com/products/metabolic-calibration-systems/3-liter/metabolic-simulator-with-mass-flow-controller/) was used to simulate human breathing and metabolism via a precise mass flow controller. Thereby different tidal volumes and respiratory rates using both the standard 2 m tube and, for comparison, the 10 m tube for MR-compatible spirometry were tested at various levels of respiratory rate and tidal volumes (Supporting Table S1). The gas flow was adjusted so that the amplitude for oxygen and carbon dioxide was ~ 3-5% difference between end-inspiration and end-expiration.

Supporting Table S1: Different levels of respiratory rate and tidal volume for the validation of the MR compatible spirometry.

level	respiratory rate (breaths/min)	tidal volume (L)
1	12	0.5
2	20	0.5
3	25	1.0
4	35	1.0
5	60	1.5
6	45	3.0

Results: Measurements of respiratory flow and respiratory gases (oxygen, carbon dioxide) using 2 m and 10 m tubing are shown in Supporting Figure S1. Respiratory flow (A), oxygen concentration (B) and carbon dioxide concentration (C) demonstrated near-identical curves for both tubing lengths (Supporting Figure S1). Only at the highest levels (5 and 6), corresponding to tidal volumes and respiratory rates typical of maximal exertion (see Supporting Table S1) [1, 2] and exceeding those observed during submaximal exercise, a slight flattening of the respiratory flow peaks and oxygen concentration was observed with the 10 m tubing (see Supporting Table S1).

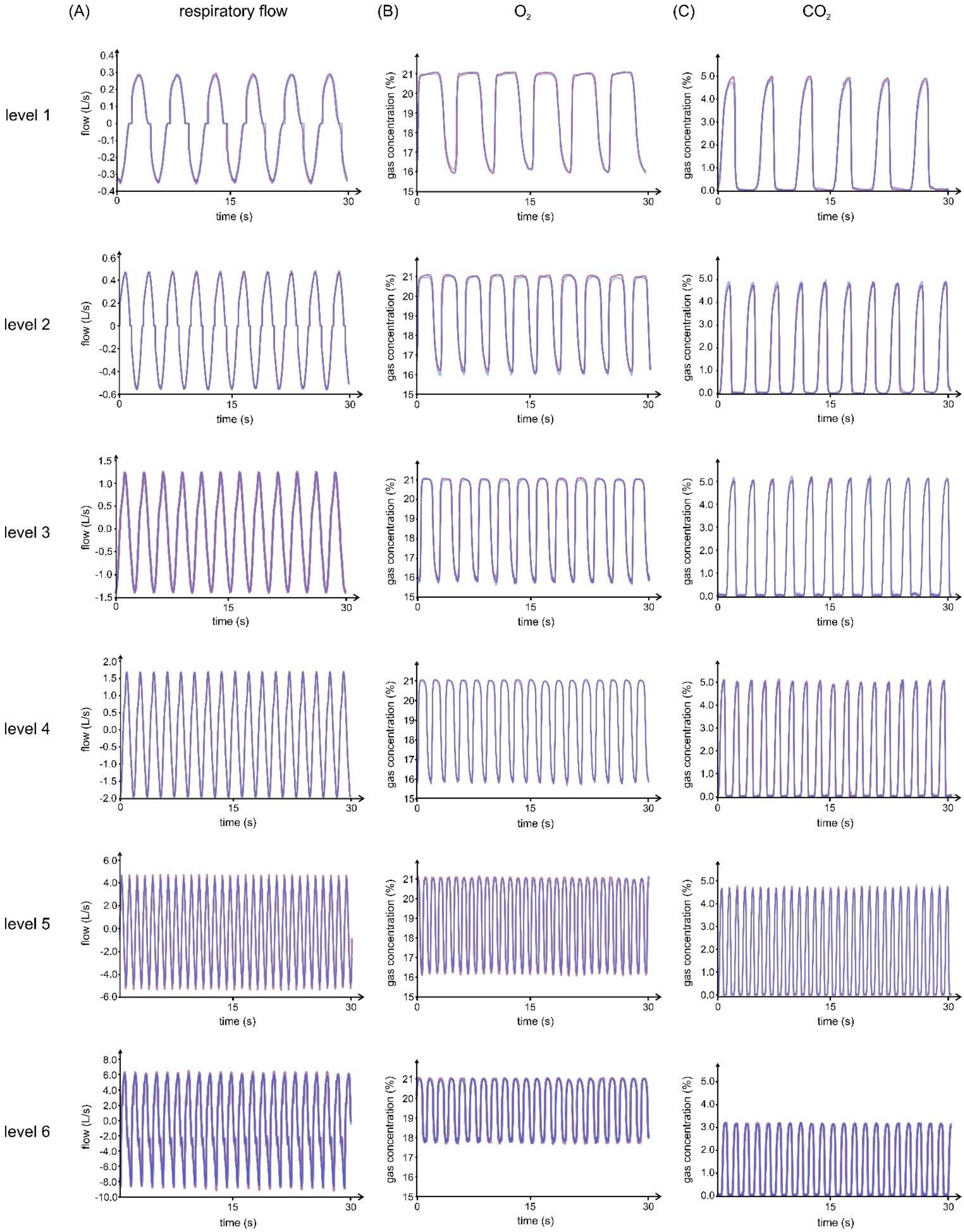

(A)
respiratory flow
(B)
O_2
(C)
CO_2
level 1
level 2
level 3
level 4
level 5
level 6
flow (L/s)
gas concentration (%)
time (s)

Supporting Figure S1 Validation of 10m long tubes.

Respiratory flow curves (A) oxygen concentration (B) and carbon dioxide concentration (C) at 6 different levels of tidal volume and respiratory rate (for details see Supporting Table S1) using the standard 2-meter tube (pink) and the 10-meter tube for MR-compatible spirometry (blue). The curves of the 2- and 10-meter tubes show an almost exact overlap. Only at the extreme level 5 and 6 (respiratory rate 45-60 breaths per minute, tidal volume1-3 L) there was a slight flattening of the maxima and minima for respiratory flow and oxygen concentration for the 10-meter tube.

Conclusion: Extending the tubing from 2 to 10 meters to allow for spirometry during an MRI scan had no significant effect on respiratory flow or respiratory gases within the expected physiological range of respiratory rate and flow during submaximal CPET.

<u>References:</u>

1. Blackie SP, Fairbarn MS, McElvaney NG, Wilcox PG, Morrison NJ, Pardy RL. Normal values and ranges for ventilation and breathing pattern at maximal exercise. Chest. 1991;100(1):136-142. doi:10.1378/chest.100.1.136.
2. Loe H, Steinshamn S, Wisløff U. Cardio-respiratory reference data in 4631 healthy men and women 20-90 years: the HUNT 3 fitness study. PLoS One. 2014;9(11):e113884. Published 2014 Nov 26. doi:10.1371/journal.pone.0113884.

Part B) Reproducibility results

Supporting Table S1: Reproducibility Analysis of Left Ventricular Cardiac Volumes

variable	ICC	conventional cine MRI	RT-MRI	
		rest		exercise
		CMR ICC		
LV-EDV (mL)	intrarater	0.99	1.00	0.99
	interrater	0.99	0.99	0.99
LV-ESV (mL)	intrarater	0.99	0.99	0.99
	interrater	0.96	0.98	0.98
LV-SV (mL)	intrarater	0.97	0.97	0.98
	interrater	0.94	0.96	0.92
LV-EF (%)	intrarater	0.95	0.91	0.96
	interrater	0.78	0.92	0.86
LV-EDMM (g)	intrarater	0.99	0.91	0.99
	interrater	0.93	0.93	0.94

Abbreviations: ICC= Intraclass correlation coefficient; LV-EDVi = left ventricular end-diastolic volume; LV-EDMM = left ventricular end-diastolic myocardial mass; LV-EF = left ventricular ejection fraction; LV-ESV= left ventricular end-systolic volume; LV-SV = left ventricular stroke volume; RT-MRI = real-time magnetic resonance imaging

Statistics: Intraclass correlation coefficient
